# Interaction and overall effects of underweight, low muscle mass, malnutrition, and inflammation on early-onset mild cognitive impairment in type 2 diabetes

**DOI:** 10.3389/fnagi.2025.1498478

**Published:** 2025-03-27

**Authors:** Chen-Ying Lin, Ya-Jie Zhai, Fan Wu, Hao-Hua An, Tong Chen, Hui-Na Qiu, Jing-Bo Li, Jing-Na Lin

**Affiliations:** ^1^Tianjin Union Medical Center, Tianjin Medical University, Tianjin, China; ^2^Department of Endocrinology, Health Management Center, Tianjin Union Medical Center, The First Affiliated Hospital of Nankai University, Tianjin, China; ^3^School of Medicine, Nankai University, Tianjin, China; ^4^Department of Clinical Laboratory, Tianjin Union Medical Center, The First Affiliated Hospital of Nankai University, Tianjin, China

**Keywords:** mild cognitive impairment, type 2 diabetes mellitus, sarcopenia, malnutrition, inflammation, interaction

## Abstract

**Introduction:**

This study systematically explores the overall impact and interactions of body composition and nutritional inflammatory indices on early-onset mild cognitive impairment (EOMCI) in type 2 diabetes mellitus (T2DM).

**Methods:**

A cross-sectional study included 816 T2DM patients. Body composition indices included body mass index (BMI), waist circumference (WC), a body shape index (ABSI), body roundness index (BRI), visceral fat area (VFA), body fat percentage (BF%), and skeletal muscle mass index (SMMI). Nutritional inflammatory indices included the geriatric nutritional risk index (GNRI), prognostic nutritional index (PNI), C-reactive protein-albumin-lymphocyte index (CALLY), and fibrinogen-to-albumin ratio (FAR). K-means clustering and quantile g-computation (QGC) assessed the combined impact, with interactions evaluated by simple slope analysis.

**Results:**

K-means clustering revealed two distinct patterns: Low-pattern and High-pattern. The Low-pattern group exhibited significantly lower body composition indices (BMI 24.6 vs. 27.7 kg/m^2^; WC 88 vs. 99 cm; ABSI 0.081 vs. 0.084; BRI 3.89 vs. 5.02; VFA 91 vs. 112; BF% 29% vs. 31%; SMMI 9.38 vs. 10.48 kg/m^2^; all *P* < 0.001) and poorer nutritional status with higher inflammation (GNRI 97.9 vs. 104.6; PNI 47.9 vs. 53.1; CALLY index 4 vs. 5; FAR 0.082 vs. 0.072; all *P* < 0.05). This group had a higher prevalence of EOMCI (32% vs. 23%, *P* = 0.006). After adjusting for confounders, the Low-pattern group had a 1.45-fold increased risk of EOMCI (OR 1.45, 95% CI 1.01–2.08). QGC analysis demonstrated that the combined overall effect of body composition and nutritional inflammatory indices was negatively associated with EOMCI risk. A one-quintile increase in all indices was linked to a significant 31.3% reduction in EOMCI risk (95% CI −44.4%, −15.0%). Interaction analysis revealed that abdominal obesity (ABSI > 0.08), combined with malnutrition (low GNRI), significantly increased EOMCI risk (*P*_*interaction*_ = 0.018). Similarly, low muscle mass (SMMI < 11.33 kg/m^2^), when combined with malnutrition and high inflammation (low CALLY index), further exacerbated EOMCI risk (*P_*interaction*_* = 0.028).

**Discussion:**

The findings suggest that in T2DM patients, the interactions and overall effects of underweight, reduced muscle mass, abdominal obesity, malnutrition, and elevated inflammation are significantly associated with an increased risk of EOMCI. Integrated management of these factors is essential to mitigate EOMCI risk.

## 1 Introduction

Over the past 30 years, the number of dementia cases in China has increased nearly fourfold, with elevated blood glucose levels recognized as one of the major risk factors (Li et al., 2022). China has the largest diabetes population globally, with approximately 116 million patients as of 2021 (GBD 2021 Diabetes Collaborators, 2023). Studies suggest that patients with midlife-onset type 2 diabetes mellitus (T2DM) have a stronger association with dementia risk compared to those with late-onset T2DM (Xu et al., 2009; Rawlings et al., 2014; Gottesman et al., 2017; Hwang et al., 2023; Hu J. et al., 2024), and the onset age of dementia is significantly earlier in this population. Notably, the age of onset for T2DM in China has shown a decreasing trend. In the past decade, new cases have rapidly increased among young and middle-aged adults (Lin et al., 2025). This trend may potentially exacerbate the dementia burden in the future.

Mild cognitive impairment (MCI) is an early stage of dementia, characterized by a decline in memory or other cognitive functions that does not meet the diagnostic criteria for dementia (Nasreddine et al., 2005). Early-onset mild cognitive impairment (EOMCI), defined as MCI occurring before the age of 65, has a prevalence of nearly 50% among individuals with diabetes (Pal et al., 2018; Makino et al., 2021; You et al., 2021). Poor glycemic control further accelerates the progression from MCI to dementia, especially within the first year after diagnosis (Pal et al., 2018; Makino et al., 2021; You et al., 2021; Ding et al., 2024). Notably, younger and middle-aged individuals have a higher potential for reversing cognitive impairment compared to older adults (Pandya et al., 2017; Overton et al., 2019; Xue et al., 2019). Therefore, early identification and intervention targeting critical risk factors for EOMCI in middle-aged patients with T2DM are crucial for delaying or reversing cognitive decline and reducing the future burden of dementia.

Recently, a prospective study based on the United Kingdom Biobank by Hendriks et al. (2024) identified multiple risk factors beyond diabetes, such as elevated C-reactive protein (CRP) and reduced grip strength, significantly associated with early-onset dementia. Numerous studies have indicated that systemic inflammation, malnutrition, and sarcopenia are closely related to cognitive decline (Roberts et al., 2009; Shen et al., 2019; Peng et al., 2020; Liu et al., 2021; Sun et al., 2021; Anita et al., 2022). Notably, these conditions frequently coexist in patients with T2DM, often accompanied by visceral fat accumulation, leading to a state known as sarcopenic obesity (Biolo et al., 2014; Gingrich et al., 2019; Ligthart-Melis et al., 2020; Izzo et al., 2021; Feng et al., 2022; Li H. et al., 2023; Ida et al., 2024; Zhang T. et al., 2024). However, previous research typically examined the association between single factors and MCI individually, ignoring their complex collinearity and interactions. Although indices such as the geriatric nutritional risk index (GNRI) (Ishihara et al., 2020; Sun et al., 2021; Xu et al., 2023), prognostic nutritional index (PNI) (Zhou et al., 2021; Wang et al., 2024), and fibrinogen-to-albumin ratio (FAR) (Li X. et al., 2023) have been widely applied in older populations to assess nutritional status and MCI risk, research in middle-aged patients with T2DM remains limited. Additionally, recently proposed indicators such as the CRP-albumin-lymphocyte (CALLY) index, reflecting both nutritional and inflammatory status (Yang et al., 2023; Li et al., 2024), and new abdominal obesity indices, such as A body shape index (ABSI) (Ji et al., 2018; Orsi et al., 2022; Lu et al., 2023) and body roundness index (BRI) (Rico-Martin et al., 2020; Wu et al., 2022), have not been extensively studied in relation to MCI risk. Therefore, it is necessary to systematically explore the combined effects and interactions of body composition, body shape indices, and nutritional-inflammation markers in middle-aged T2DM patients to comprehensively understand their impact on EOMCI risk.

This study adopted a cross-sectional design focusing on middle-aged Chinese patients with T2DM. We employed K-means clustering to identify distinct patterns of body composition, body shape, and nutritional-inflammation indices. Quantile g-computation (QGC) was applied to systematically explore the combined effects of these indices on the risk of EOMCI. Subsequently, we utilized least absolute shrinkage and selection operator (LASSO) regression to further identify key risk factors and performed simple slope analyses to examine potential interactions among these indices. This study aims to clarify the complex associations between body composition, nutritional-inflammation status, and EOMCI risk, thus providing theoretical support for early screening and precise interventions to delay EOMCI progression and reduce the long-term dementia burden.

## 2 Materials and methods

### 2.1 Study population and cognitive assessment

This study is an observational cross-sectional study involving patients who first visited the Endocrinology Department of Nankai University Affiliated Hospital between July 2018 and February 2024. Cognitive function was screened using the Montreal Cognitive Assessment (MoCA), which has been proven to be a convenient and sensitive tool for detecting MCI (Nasreddine et al., 2005; Lu et al., 2011). All participants underwent their initial MoCA test administered by trained professional doctors. To adjust for the impact of educational years on MoCA scores, we added one point to the total MoCA score for participants with 12 or fewer years of education if their score was less than 30 (Nasreddine et al., 2005). According to the MoCA norms reported by Lu et al. (2011) for mainland China, participants were diagnosed with MCI if their MoCA scores were ≤13 for illiterate individuals, ≤19 for those with 1–6 years of education, and ≤24 for those with seven or more years of education.

### 2.2 Participants selection

Inclusion criteria were patients aged 65 years or younger with diabetes diagnosed per the 1999 WHO criteria. Exclusion criteria included type 1, gestational, or other specific types of diabetes; inability to complete bioelectrical impedance analysis (BIA) due to severe edema, implanted metal devices, pregnancy, or skin damage; missing key variables (albumin, CRP, lymphocyte count, or fibrinogen); inability to complete the MoCA test due to visual or hearing impairments; a history of cerebrovascular diseases, severe head trauma, mental illness, or neurological disorders; renal failure [eGFR < 60 mL/min/1.73 m^2^ or urine albumin-to-creatinine ratio (UACR) > 30 mg/mmol]; heart failure; liver dysfunction [aspartate aminotransferase (AST) and/or alanine aminotransferase (ALT) ≥2 times the upper limit]; diabetic ketoacidosis, ketonuria, or infections; malignancies, autoimmune diseases, severe anemia [hemoglobin (Hb) < 60 g/L]; and thyroid dysfunction. A total of 816 T2DM patients under 65 years were included: 218 diagnosed with EOMCI and 598 in the non-MCI control group (NOMCI). The participant selection flowchart is shown in Supplementary Figure 1.

### 2.3 Data collection

This study collected participants’ demographic characteristics (sex, age, education level, marital status), lifestyle factors (smoking status, drinking status, regular exercise, diabetes dietary control), diabetes duration, hypoglycemia frequency in the past 3 months, medical history, and medication use through standardized questionnaires, face-to-face interviews, and medical record reviews. Comprehensive evaluations were conducted for complications and comorbidities, including dyslipidemia, diabetic microvascular complications (DMC), peripheral arterial atherosclerosis (PAA), and coronary heart disease (CHD). All data were collected by trained healthcare professionals, who were only involved in data collection. Blood cell counts, Hb levels, serum biochemistry, and urinary parameters were measured using standardized methods. Written informed consent was obtained from all participants. The study was conducted strictly in accordance with the ethical principles outlined in the Declaration of Helsinki. Ethical approval was granted by the Ethics Committee of Tianjin Union Medical Center [Approval No. 2018 (C08)]. Detailed methods for data collection, laboratory measurements, and variable definitions are provided in Supplementary materials.

### 2.4 Anthropometry and body composition analysis

In this study, participants’ height and weight were measured using an automated height-weight measuring device while they stood barefoot, wore light clothing, and had an empty bladder. Waist circumference (WC) was measured at the midpoint between the lower rib margin and the iliac crest using a flexible tape measure. Body composition was assessed using a body composition analyzer (InBody770, Biospace, South Korea) through BIA, which evaluated skeletal muscle mass, body fat percentage (BF%), and visceral fat area (VFA). The body composition indices used in this study included body mass index (BMI), WC, ABSI, BRI, skeletal muscle mass index (SMMI), BF%, and VFA. The formulas for these calculations are as follows:

(1) BMI = weight (kg)/height^2^ (m^2^)
(2) $\text{ABSI}=\frac{\text{WC}}{{\text{BMI}}^{2/3}\times {\text{height}}^{1/2}}$, where WC and height are in meters (Krakauer and Krakauer, 2012).
(3) $\text{BRI}=364.2-365.5\times \sqrt{1-\left(\frac{{\left(\frac{\text{WC }}{2\,\pi }\right)}^{2}}{{\left(0.5\times \text{height}\right)}^{2}}\right)}$, where WC and height are in cm (Chang et al., 2015).
(4) SMMI = skeletal muscle mass (kg)/height^2^ (m^2^) (Lee et al., 2020).

### 2.5 Nutritional and inflammatory indices

The nutritional and inflammatory indices used in this study included the GNRI, PNI, CALLY index, and FAR. Lower GNRI and PNI values indicate poorer nutritional status. A lower CALLY index suggests malnutrition accompanied by a high inflammation state, while a higher FAR indicates higher inflammation and poorer nutritional status. The formulas for these calculations are as follows:

(1) $\text{GNRI}=\left[1.489\times \mathrm{albumin}\,\left(g/L\right)\right]+\left[41.7\times \left(\frac{\text{weight}\,\left(\text{kg}\right)}{\text{WLO}}\right)\right];$$\text{WLO}=\text{height}\,\left(\text{cm}\right)-100-\frac{\left[\text{height}\,\left(\text{cm}\right)-150\right]}{4}$ for male; $\text{WLO}=\text{height}\,\left(\text{cm}\right)-100-\frac{\left[\text{height}\,\left(\text{cm}\right)-150\right]}{2.5}$ for female (Bouillanne et al., 2005)
(2) PNI = albumin(g/L) + lymphocytes (× 10^9^/L) × 5 (Pinato et al., 2012)
(3) $\mathrm{CALLY}\,\mathrm{index}=\frac{\mathrm{albumin}\,\left(g/\mathrm{dL}\right)\times \mathrm{lymphocyte}\,\mathrm{count}\,\left(\mu \,\text{L}\right)}{\text{CRP}\,\left(\text{mg}/\text{dL}\right)\times 10^{4}}$ (Tsai et al., 2022)
(4) $\text{FAR}=\frac{\mathrm{fibrinogen}\,\left(g/L\right)}{\mathrm{albumin}\,\left(g/L\right)}$ (Wang et al., 2021)

### 2.6 Statistical analysis

We compared baseline covariates, body composition, and nutritional inflammatory indices between the EOMCI and NOMCI groups. Continuous variables were tested for normality using the Kolmogorov-Smirnov test and Q-Q plots. Normally distributed variables were presented as mean and standard deviation (SD) and compared using Student’s *t*-test. Non-normally distributed variables were expressed as median and interquartile range (IQR) and compared with the Wilcoxon rank sum test. Categorical variables were reported as frequencies and percentages and compared using the Chi-squared or Fisher’s exact test. Pearson correlation analysis was performed for body composition and nutritional inflammatory indices, and correlations were visualized with a heatmap.

To evaluate independent associations between body composition, nutritional inflammatory indices, and EOMCI, we performed multivariable logistic regression analyses. Each index was divided into tertiles, with the third tertile as the reference. Three models were created: Model 1 was unadjusted; Model 2 adjusted for sociodemographic factors (age, sex, marital status, education); and Model 3 further adjusted for diabetes-related variables (duration, HbA1c, fasting plasma glucose (FPG), hypoglycemia frequency) and baseline covariates with significant differences (smoking, regular exercise, dietary control, UACR, AST, and Hb).

To examine clustering patterns of body composition and nutritional inflammatory status and their overall effect on EOMCI, we performed K-means clustering. All variables were standardized using Z-scores. Specifically, each observed value of a variable was adjusted by subtracting the variable’s mean and then dividing by its standard deviation. This ensured that the distribution of each variable had a mean of zero and a standard deviation of one, giving all variables equal weight in the clustering analysis. The elbow method identified two optimal clusters, and 25 random initializations were conducted. We then compared the prevalence of EOMCI, MoCA scores, and baseline differences between these clusters. Multivariable logistic regression was used to assess the independent association between clustering patterns and EOMCI, adjusting for potential confounders, consistent with the previous logistic regression model settings.

Quantile g-computation (QGC) analysis is a statistical method used to evaluate the relationship between mixed exposures and health outcomes. QGC divides exposure variables into quantiles, estimates the effect of each quantile using a generalized linear model (GLM), and then calculates the weighted average of the effects to obtain the overall effect of the mixed exposures on health outcomes. This method addresses the high dimensionality and multicollinearity issues among body composition and nutritional inflammatory indices, providing an overall effect estimate. In this study, we transformed the indices into quintiles and estimated the percentage change in EOMCI risk for a simultaneous one-quintile increase in all indices as (*e*^β^−1) × 100%. QGC also assessed the positive and negative weights of each index, indicating their impact on EOMCI risk and relative contribution. We constructed two models and used 1,000 bootstrap resampling to ensure the robustness of the results: Model 1 was unadjusted, and Model 2 adjusted for confounders (sociodemographic factors, diabetes-related variables, and baseline significant variables). Additionally, we performed stratified analyses to compare the overall effect of body composition and nutritional inflammatory status on EOMCI across different subgroups based on sex, education level, diabetes duration, HbA1c tertiles, hypoglycemia frequency in the past 3 months, DMC, PAA, and CHD.

In addition to QGC analysis, which evaluates the relative contribution of each index to the risk of EOMCI through their positive and negative weights, we employed the Least Absolute Shrinkage and Selection Operator (LASSO) regression. LASSO adds an L1 regularization term to the loss function, effectively shrinking some regression coefficients to zero, thus enabling variable selection. LASSO is particularly effective in identifying key indices influencing EOMCI in the presence of multicollinearity. In this study, LASSO regression combined with ten-fold cross-validation was used for variable selection. Key indices were selected based on two regularization parameters: one that minimizes the mean squared error (MSE) (Min standard) and another that is within one standard error of the minimum MSE (1SE standard).

To further evaluate their interaction effects on EOMCI, we constructed logistic regression models with multiplicative interaction terms, adjusting comprehensively for confounders. To visualize these interactions, we created interaction plots with nutritional inflammatory indices as primary predictors and body composition indices as moderators, grouped into tertiles. Additionally, we performed simple slope analysis to explore the effects of GNRI and CALLY on EOMCI at different levels of BMI, ABSI, and SMMI. Johnson-Neyman plots were generated to identify critical values of body composition indices, showing confidence intervals where the effect of nutritional inflammatory indices on EOMCI is significant.

All statistical analyses were conducted using R software (version 4.3.2). QGC and interaction analyses were performed with the “qgcomp” and “interactions” packages, respectively. Tests were two-sided, with *P* < 0.05 considered significant. The statistical power of the fully adjusted logistic regression model (Model 3) was calculated using the “pwr” package. With an effect size (Cohen’s f^2^) of 0.15, a significance level of 0.05, a sample size of 816, and 15 predictors, the power was 1.0. A similar power analysis for the *t*-test used in baseline comparisons also showed a power of 1.0.

## 3 Results

### 3.1 Baseline characteristics of participants

Table 1 presents the baseline characteristics of the 816 participants, with an EOMCI prevalence of 27%. Compared to the NOMCI group, the EOMCI group had a significantly higher average age (59.2 vs. 57.4 years, *P* < 0.001), a lower proportion of participants with more than 12 years of education (10% vs. 18%, *P* = 0.003), and a higher proportion of unmarried individuals (12% vs. 4.3%, *P* < 0.001). While there were no significant differences in drinking status, the EOMCI group had a higher proportion of current or former smokers (51% vs. 46%, *P* = 0.004), a higher proportion of participants engaging in regular exercise (79% vs. 71%, *P* = 0.023), and a higher likelihood of not following diabetes dietary control (43% vs. 33%, *P* = 0.013). The EOMCI group also had a significantly higher frequency of hypoglycemia episodes in the past 3 months (*P* = 0.017), but there were no significant differences in diabetes duration, antidiabetic medication use, FPG, or HbA1c. Additionally, there were no significant differences between the two groups in terms of diabetes complications or comorbidities.

**TABLE 1:** Baseline characteristics of participants stratified by cognitive status.

Characteristic	Overall, *N* = 816 (100%)	NOMCI, *N* = 598 (73%)	EOMCI, *N* = 218 (27%)	*P*-value
Age	57.9 (5.1)	57.4 (5.3)	59.2 (4.3)	<0.001*
Sex-females	367 (45%)	260 (43%)	107 (49%)	0.154
Duration of education				0.003*
<9 years	47 (5.8%)	40 (6.7%)	7 (3.2%)	
9–12 years	642 (79%)	453 (76%)	189 (87%)	
> 12 years	127 (16%)	105 (18%)	22 (10%)	
Unmarried	52 (6.4%)	26 (4.3%)	26 (12%)	<0.001*
Smoking status				0.004*
Current smoker	277 (34%)	209 (35%)	68 (31%)	
Former smoker	111 (14%)	67 (11%)	44 (20%)	
Never smoker	428 (52%)	322 (54%)	106 (49%)	
Drinking status				0.085
Current drinker	265 (32%)	197 (33%)	68 (31%)	
Former drinker	62 (7.6%)	38 (6.4%)	24 (11%)	
Never drinker	489 (60%)	363 (61%)	126 (58%)	
Regular exercise	596 (73%)	424 (71%)	172 (79%)	0.023*
Non-dietary control	292 (36%)	199 (33%)	93 (43%)	0.013*
Duration of diabetes	5 (1, 10)	5 (1, 10)	5 (1, 11)	0.461
Hypoglycemia frequency				0.017*
0 times	653 (80%)	493 (82%)	160 (73%)	
1–2 times	141 (17%)	91 (15%)	50 (23%)	
≥3 times	22 (2.7%)	14 (2.3%)	8 (3.7%)	
Antidiabetic agents				
Metformin-yes	336 (41%)	255 (43%)	81 (37%)	0.159
Other oral drugs-yes	494 (61%)	357 (60%)	137 (63%)	0.416
Insulin-yes	243 (30%)	172 (29%)	71 (33%)	0. 293
GLP-1RA-yes	13 (1.6%)	11 (1.8%)	2 (0.9%)	0.531
DMC-yes	661 (81%)	484 (81%)	177 (81%)	0.934
PAA-yes	552 (68%)	397 (66%)	155 (71%)	0.203
CHD-yes	197 (24%)	147 (25%)	50 (23%)	0.627
Dyslipidemia-yes	450 (55%)	336 (56%)	114 (52%)	0.322
FPG (mmol/L)	8.28 (6.69, 10.52)	8.30 (6.82, 10.53)	8.16 (6.47, 10.48)	0.250
HbA1C (%)	8.55 (7.40, 10.40)	8.70 (7.50, 10.40)	8.30 (7.20, 10.30)	0.070
Uric acid (μmol/L)	291 (80)	290 (79)	291 (84)	0.956
UACR (mg/mmol)	1 (1, 4)	1 (1, 4)	2 (1, 6)	0.019*
eGFR (mL/min/1.73 m^2^)	125 (28)	126 (26)	124 (32)	0.367
GGT (U/L)	27 (20, 41)	27 (20, 42)	27 (18, 39)	0.203
ALT U/L)	20 (15, 31)	20 (15, 31)	18 (14, 31)	0.104
AST (U/L)	18 (14, 23)	17 (14, 23)	19 (15, 23)	0.038*
Hb (g/L)	138 (15)	138 (15)	135 (16)	0.020*
WBC count (10^9^/L)	6.03 (5.05, 7.18)	6.04 (5.05, 7.18)	6.01 (5.10, 7.23)	0.899
SBP (mmHg)	132 (13)	132 (13)	133 (13)	0.512
DBP (mmHg)	80 (8)	80 (8)	80 (8)	0.850
BMI (kg/m^2^)	26.3 (3.7)	26.6 (3.7)	25.7 (3.6)	0.002*
WC (cm)	94 (11)	95 (12)	93 (11)	0.020*
ABSI	0.083 (0.004)	0.082 (0.004)	0.083 (0.004)	0.256
BRI	4.53 (3.75, 5.46)	4.59 (3.78, 5.58)	4.41 (3.72, 5.31)	0.086
VFA	102 (31)	103 (32)	101 (30)	0.291
BF%	30 (8)	30 (8)	31 (8)	0.731
SMMI (kg/m^2^)	10.00 (1.51)	10.11 (1.50)	9.68 (1.50)	<0.001*
GNRI	101.7 (5.6)	102.1 (5.2)	100.4 (6.5)	<0.001*
PNI	50.8 (5.1)	51.2 (4.9)	49.9 (5.4)	0.002*
CALLY index	4 (2, 9)	5 (3, 10)	3 (1, 6)	<0.001*
FAR	0.076 (0.066, 0.089)	0.075 (0.065, 0.088)	0.077 (0.068, 0.091)	0.107

Mean (standard deviation, SD) for normally distributed continuous variables and compared using Student’s *t*-test; median (interquartile ranges, IQRs) for skewed continuous variables and compared using Wilcoxon rank sum test; *n* (%) for categorical variables and compared using Chi-squared test or Fisher’s exact test;

*P < 0.05. NOMCI, non-mild cognitive impairment; EOMCI, early-onset mild cognitive impairment; GLP-1RA, glucagon-like peptide-1 receptor agonist; DMC, diabetic microvascular complications; PAA, peripheral arterial atherosclerosis; CHD, coronary heart disease; FPG, fasting plasma glucose; HbA1C, hemoglobin A1C; UACR, urine albumin-to-creatinine ratio; eGFR, estimated glomerular filtration rate; GGT, gamma-glutamyl transferase; AST, aspartate aminotransferase; ALT, alanine aminotransferase; Hb, hemoglobin; WBC, white blood cell; SBP/DBP, systolic/diastolic blood pressure; BMI, body mass index; WC, waist circumference; ABSI, A body shape index; BRI, body roundness index; VFA, visceral fat area; BF%, body fat percentage; SMMI, skeletal muscle mass index; GNRI, geriatric nutritional risk index; PNI, prognostic nutritional index; CALLY, C-reactive protein-albumin-lymphocyte index; FAR, fibrinogen to albumin ratio.

In laboratory test indicators, the EOMCI group had significantly higher UACR levels (2 vs. 1, *P* = 0.019), higher AST levels (19 vs. 17, *P* = 0.038), and lower Hb levels (135 vs. 138, *P* = 0.020). Regarding body composition, the EOMCI group had significantly lower BMI, WC, and SMMI compared to the NOMCI group (all *P* < 0.05), but no significant differences in ABSI, BRI, VFA, or BF% were observed. In terms of nutritional inflammatory indices, the EOMCI group had significantly lower GNRI, PNI, and CALLY indices (all *P* < 0.05), while FAR did not differ significantly between the two groups (*P* = 0.107).

### 3.2 Independent association between individual body composition and nutritional inflammatory indices with EOMCI

**FIGURE 1:**
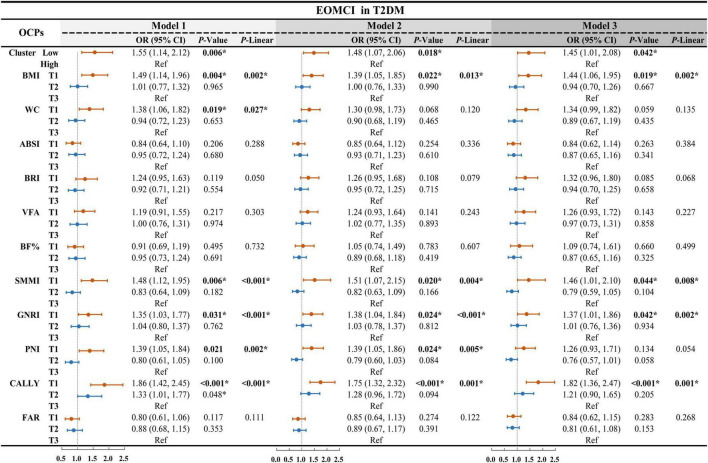
Multivariable-adjusted logistic regression for body composition, nutritional inflammatory indices, and cluster patterns on EOMCI in T2DM. EOMCI, early-onset mild cognitive impairment; T2DM, type 2 diabetes mellitus; BMI, body mass index; WC, waist circumference; ABSI, A body shape index; BRI, body roundness index; VFA, visceral fat area; BF%, body fat percentage; SMMI, skeletal muscle mass index; GNRI, geriatric nutritional risk index; PNI, prognostic nutritional index; CALLY, C-reactive protein-albumin-lymphocyte index; FAR, fibrinogen to albumin ratio; OR, odds ratio; CI, confidence interval; Ref, reference. This forest plot presents multivariable-adjusted logistic regression analyses examining the associations between individual body composition indices, nutritional inflammatory indices, and their cluster patterns with the risk of EOMCI in participants with T2DM. The analyses include three models: Model 1 (unadjusted), Model 2 (adjusted for age, sex, marital status, and duration of education), and Model 3 (further adjusted for diabetes duration, HbA1c, FPG, hypoglycemia frequency, smoking status, regular exercise, diabetes dietary control, UACR, AST, and Hb). *P < 0.05; *P*-Linear, the *P*-value for the linear trend test.

To investigate the independent association between individual body composition and nutritional inflammatory indices with EOMCI, we conducted multivariable-adjusted logistic regression analyses, as shown in Figure 1. In the unadjusted Model 1, participants in the lowest tertile (T1) of BMI, WC, and SMMI had a significantly higher risk of EOMCI compared to those in the highest tertile (T3). The increased risk associated with the lowest tertile of BMI and SMMI remained significant in Model 2, which adjusted for sociodemographic factors, and in Model 3, which further adjusted for potential confounders. In Model 3, the odds ratios (ORs) were 1.44 (95% CI 1.06–1.95) for BMI and 1.46 (95% CI 1.01–2.10) for SMMI.

For nutritional inflammatory indices, the unadjusted Model 1 showed that participants in the lowest tertile of GNRI, PNI, and CALLY had a significantly higher risk of EOMCI compared to those in the highest tertile. After full adjustment in Model 3, only the lowest tertiles of GNRI and CALLY remained significantly associated with increased EOMCI risk. Specifically, participants in the lowest tertile of GNRI had a 1.37 times higher risk of EOMCI (OR = 1.37, 95% CI 1.01–1.86), and those in the lowest tertile of CALLY had a 1.82 times higher risk (OR = 1.82, 95% CI 1.36–2.47) compared to those in the highest tertile.

### 3.3 Correlation analysis and clustering patterns of body composition and nutritional inflammatory indices

**FIGURE 2:**
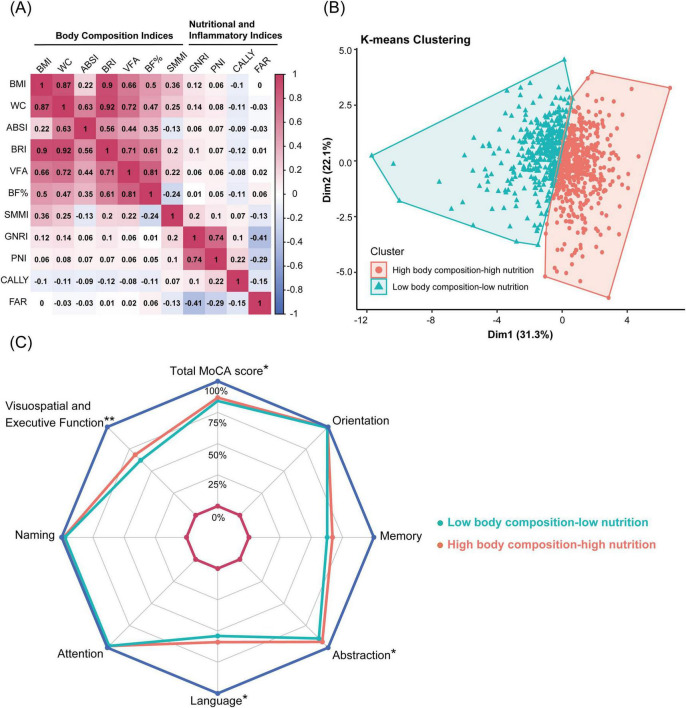
Correlation analysis and K-means clustering of body composition and nutritional inflammatory indices. **(A)** Heatmap showing the Pearson correlation matrix of body composition and nutritional inflammatory indices. **(B)** K-means clustering of 816 participants based on body composition and nutritional inflammatory indices, identifying two clusters: high body composition-high nutrition pattern and low body composition-low nutrition pattern. **(C)** Radar chart comparing total MoCA scores and subdomain scores between the high body composition-high nutrition pattern and the low body composition-low nutrition pattern. Significant differences are marked: **P < 0.001, *P < 0.05. BMI, body mass index; WC, waist circumference; ABSI, A body shape index; BRI, body roundness index; VFA, visceral fat area; BF%, body fat percentage; SMMI, skeletal muscle mass index; GNRI, geriatric nutritional risk index; PNI, prognostic nutritional index; CALLY index, C-reactive protein-albumin-lymphocyte index; FAR, fibrinogen to albumin ratio.

Figure 2A shows the complex relationships between body composition and nutritional inflammatory indices. Most body composition indices were strongly correlated, whereas ABSI and SMMI showed weaker or moderate correlations with other body composition indices. Similarly, various nutritional inflammatory indices exhibited different degrees of correlation.

K-means clustering analysis was employed to identify subgroups with similar characteristics. Based on the body composition and nutritional inflammatory indices of 816 participants, two clustering patterns were identified: a high body composition-high nutrition pattern (High-pattern, 56%) and a low body composition-low nutrition pattern (Low-pattern, 44%). Participants in the Low-pattern had significantly lower BMI, WC, ABSI, BRI, VFA, BF%, and SMMI compared to those in the High-pattern (all *P* < 0.001). Additionally, the Low-pattern group had significantly lower GNRI, PNI, and CALLY indices and higher FAR, indicating poorer nutritional status and higher inflammation levels (all *P* < 0.05) (Table 2 and Figure 2B).

**TABLE 2:** Baseline characteristics of K-means clustering patterns of body composition and nutritional inflammatory indices.

Indices	Overall, *N* = 816 (100%)	High-pattern, *N* = 458 (56%)	Low-pattern, *N* = 358 (44%)	*P-*value
BMI (kg/m^2^)	26.3 (3.7)	27.7 (3.6)	24.6 (3.0)	< 0.001*
WC (cm)	94 (11)	99 (11)	88 (9)	< 0.001*
ABSI	0.083 (0.004)	0.084 (0.004)	0.081 (0.004)	< 0.001*
BRI	4.53 (3.75, 5.46)	5.02 (4.27, 5.97)	3.89 (3.32, 4.62)	< 0.001*
VFA	102 (31)	112 (31)	91 (27)	< 0.001*
BF%	30 (8)	31 (7)	29 (8)	< 0.001*
SMMI (kg/m^2^)	10.00 (1.51)	10.48 (1.45)	9.38 (1.38)	< 0.001*
GNRI	101.7 (5.6)	104.6 (4.0)	97.9 (5.0)	< 0.001*
PNI	50.8 (5.1)	53.1 (4.2)	47.9 (4.5)	< 0.001*
CALLY index	4 (2, 9)	5 (2, 9)	4 (2, 8)	0.005*
FAR	0.076 (0.066, 0.089)	0.072 (0.062, 0.082)	0.082 (0.072, 0.099)	< 0.001*
EOMCI	218 (27%)	105 (23%)	113 (32%)	0.006*

Data are presented as mean (standard deviation, SD) for normally distributed continuous variables and compared using Student’s *t*-test; median (interquartile ranges, IQRs) for skewed continuous variables and compared using the Wilcoxon rank sum test; *n* (%) for categorical variables and compared using the Chi-squared test. Significant differences are marked: *P < 0.05. High-pattern, high body composition-high nutrition pattern; Low-pattern, low body composition-low nutrition pattern; EOMCI, early-onset mild cognitive impairment; BMI, body mass index; WC, waist circumference; ABSI, A body shape index; BRI, body roundness index; VFA, visceral fat area; BF%, body fat percentage; SMMI, skeletal muscle mass index; GNRI, geriatric nutritional risk index; PNI, prognostic nutritional index; CALLY index, C-reactive protein-albumin-lymphocyte index; FAR, fibrinogen to albumin ratio.

The prevalence of EOMCI was higher in the Low-pattern group compared to the High-pattern group (32% vs. 23%, *P* = 0.006) (Table 2). The Low-pattern group also had significantly lower MoCA total scores and sub-scores in visuospatial and executive function, abstraction, and language (all *P* < 0.05) (Figure 2C). Multivariable-adjusted logistic regression analysis showed that, after fully adjusting for confounders in Model 3, the Low-pattern was associated with a 1.45 times higher risk of EOMCI compared to the High-pattern (OR 1.45, 95% CI 1.01–2.08) (Figure 1).

### 3.4 Quantile g-computation for assessing the overall effect of body composition and nutritional inflammatory indices

**FIGURE 3:**
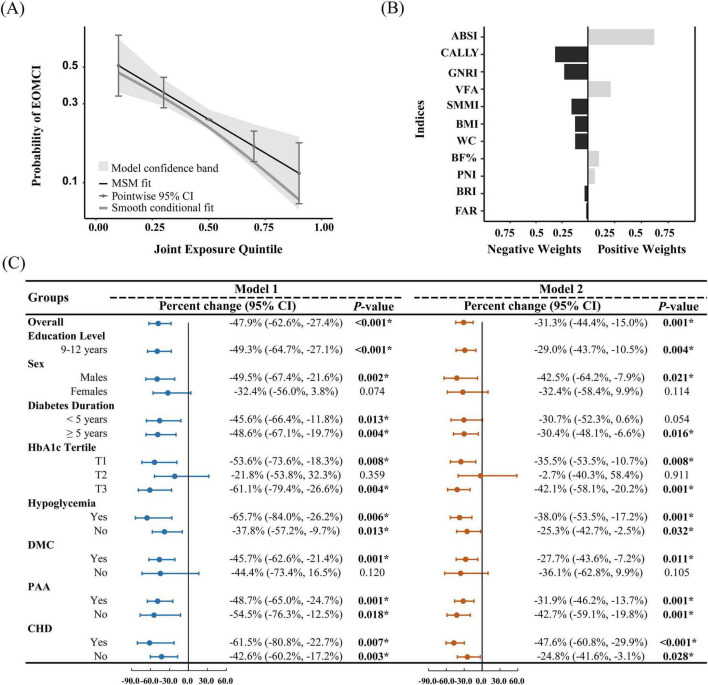
Quantile g-computation for assessing the overall effect of body composition and nutritional inflammatory indices, and the positive and negative contributions of indices. **(A)** Probability of EOMCI by joint exposure quintile, with the third quintile as the reference. **(B)** Positive and negative weights of body composition and nutritional inflammatory indices on EOMCI risk, based on the fully adjusted Model 2. **(C)** Overall analysis and stratified analysis by sex, education level, diabetes duration, HbA1c tertiles, hypoglycemia, DMC, PAA, and CHD. The figure shows the percentage change and 95% confidence intervals (CIs) for the overall effect of body composition and nutritional inflammatory indices on EOMCI risk. Model 1 (unadjusted) and Model 2 (adjusted for age, sex, marital status, education level, diabetes duration, HbA1c, FPG, hypoglycemia frequency, smoking status, regular exercise, diabetes dietary control, UACR, AST, and Hb, with respective adjustments for each subgroup, excluding the stratifying variable). Significant differences are marked: *P < 0.05. BMI, body mass index; WC, waist circumference; ABSI, A body shape index; BRI, body roundness index; VFA, visceral fat area; BF%, body fat percentage; SMMI, skeletal muscle mass index; GNRI, geriatric nutritional risk index; PNI, prognostic nutritional index; CALLY, C-reactive protein-albumin-lymphocyte index; FAR, fibrinogen to albumin ratio; DMC, diabetic microvascular complications; PAA, peripheral arterial atherosclerosis; CHD, coronary heart disease.

We also utilized QGC analysis to estimate the overall effect of body composition and nutritional inflammatory status on EOMCI. As shown in Figures 3A, C and both before and after adjusting for confounders, body composition and nutritional inflammatory indices were generally negatively associated with the risk of EOMCI. In the fully adjusted Model 2, a one-quintile increase in all indices was associated with a significant 31.3% reduction in EOMCI risk (95% CI −44.4%, −15.0%). Figure 3B further illustrates the positive and negative weights of each index on EOMCI risk. In the model adjusted for confounders, CALLY had the highest negative impact weight at 31.6%, followed by GNRI (22.9%), SMMI (15.9%), and BMI (12.4%). Conversely, ABSI had the highest positive impact weight at 61.9%.

Stratified analysis in Figure 3C further revealed the overall effect of body composition and nutritional inflammatory indices across different subgroups. The negative association remained significant among participants with 9–12 years of education. In sex-stratified analysis, the negative association was significant in males but not in females. Participants with a diabetes duration of more than 5 years showed a significant negative association after adjustment. Significant negative associations were observed in the lowest (T1) and highest (T3) tertiles of HbA1c but not in the middle tertile (T2). Significant negative associations were also found in subgroups with hypoglycemia, CHD, and PAA. Only participants with DMC exhibited a significant negative association, whereas no significant association was found in those without DMC.

#### 3.5 LASSO cross-validation for key indices selection

**FIGURE 4:**
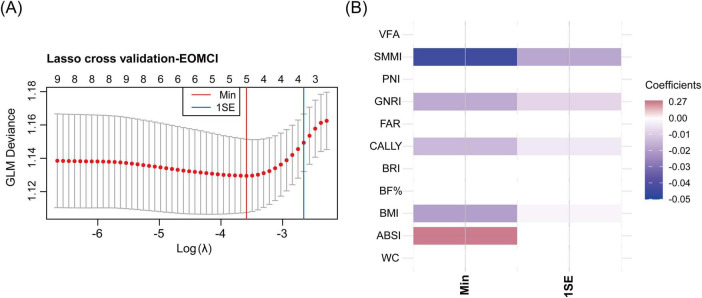
LASSO cross-validation for identifying key body composition and nutritional inflammatory indices associated with EOMCI. **(A)** LASSO cross-validation analysis for selecting key indices, showing the optimal λ (lambda) values using the minimum criteria (Min) and the 1 standard error (1SE) criteria. **(B)** Heatmap of coefficients of the selected indices under Min and 1SE criteria. EOMCI, early-onset mild cognitive impairment; BMI, body mass index; WC, waist circumference; ABSI, A body shape index; BRI, body roundness index; VFA, visceral fat area; BF%, body fat percentage; SMMI, skeletal muscle mass index; GNRI, geriatric nutritional risk index; PNI, prognostic nutritional index; CALLY, C-reactive protein-albumin-lymphocyte index; FAR, fibrinogen to albumin ratio.

Using the Min criterion, five key indices were identified through LASSO cross-validation: SMMI, GNRI, CALLY index, BMI, and ABSI. Consistent with the QGC analysis, SMMI, GNRI, CALLY index, and BMI were negatively associated with the risk of EOMCI, while ABSI was positively associated with EOMCI risk. Using the 1SE criterion, only indices negatively associated with EOMCI risk were selected as key indices (Figure 4).

### 3.6 Interaction between key body composition and nutritional inflammatory indices

**FIGURE 5:**
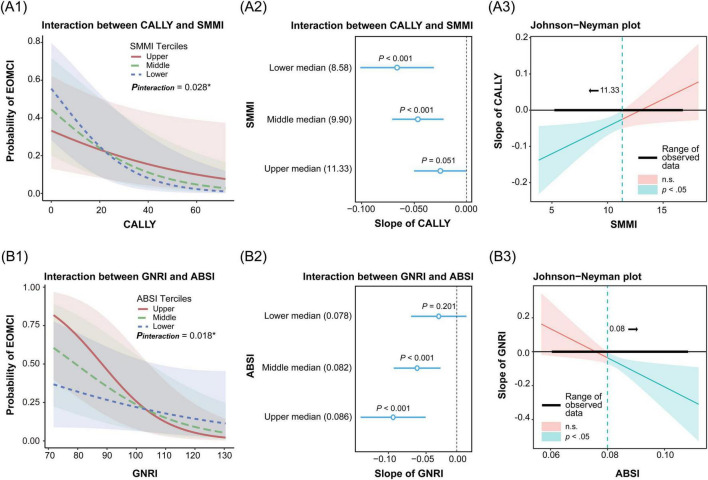
Interaction and simple slopes analysis of body composition and nutritional inflammatory indices on the risk of early-onset mild cognitive impairment (EOMCI). **(A1–A3)** Interaction between C-reactive protein-albumin-lymphocyte index (CALLY) and skeletal muscle mass index (SMMI) on EOMCI risk. **(A1)** Interaction effect of CALLY and SMMI across SMMI tertiles. (**A2)** Simple slopes of CALLY on EOMCI risk at SMMI tertiles. **(A3)** Johnson-Neyman plot showing the significance region of CALLY’s effect on EOMCI risk across SMMI values. **(B1–B3)** Interaction between geriatric nutritional risk index (GNRI) and A body shape index (ABSI) on EOMCI risk. **(B1)** Interaction effect of GNRI and ABSI across ABSI tertiles. **(B2)** Simple slopes of GNRI on EOMCI risk at different ABSI tertiles. **(B3)** Johnson-Neyman plot showing the significance region of GNRI’s effect on EOMCI risk across ABSI values. All analyses are based on generalized linear models and adjusted for age, sex, marital status, education level, diabetes duration, HbA1c, FPG, hypoglycemia frequency, smoking status, regular exercise, diabetes dietary control, UACR, AST, and Hb.

Through QGC analysis and LASSO cross-validation, we identified key body composition indices (BMI, ABSI, and SMMI) and nutritional inflammatory indices (GNRI and CALLY). To evaluate their interaction effects on EOMCI, we conducted analyses using GNRI and CALLY as predictors and BMI, ABSI, and SMMI as moderators. Significant interactions were observed between CALLY and SMMI, and between GNRI and ABSI (Figure 5), while no significant interactions were found for other combinations (Supplementary Figure 2).

Figure 5A1 shows a significant interaction between CALLY and SMMI (*P* = 0.028). When both SMMI and CALLY indices were lower, the risk of EOMCI was higher. Figure 5A2 further shows that the negative association between CALLY and EOMCI risk was most pronounced in the lowest tertile of SMMI (slope = −0.070, 95% CI −0.101, −0.031). It was followed by the middle tertile (slope = −0.047, 95% CI −0.071, −0.021). No significant association was observed in the highest tertile (slope = −0.025, 95% CI −0.050, 0.0002). The Johnson-Neyman plot in Figure 5A3 indicates that the negative impact of CALLY on EOMCI risk is significant (*P* < 0.05) when SMMI is less than 11.33. However, this effect is not significant when SMMI exceeds 11.33. This indicates that low SMMI combined with a low CALLY index, reflecting malnutrition and high inflammation, significantly increases the risk of EOMCI.

Figure 5B1 shows a significant interaction between GNRI and ABSI (*P* = 0.018). When ABSI was higher and GNRI was lower, the risk of EOMCI increased. Figure 5B2 shows that the negative association between GNRI and EOMCI risk was most pronounced in the highest tertile of ABSI (slope = −0.091, 95% CI −0.137, −0.044). It was followed by the middle tertile (slope = −0.057, 95% CI −0.090, −0.023). No significant association was observed in the lowest tertile (slope = −0.026, 95% CI −0.065, 0.014). The Johnson-Neyman plot in Figure 5B3 shows that the negative impact of GNRI on EOMCI risk is significant (*P* < 0.05) when ABSI is greater than 0.08. However, this effect is not significant when ABSI is less than 0.08. This indicates that high ABSI combined with low GNRI, reflecting malnutrition, significantly increases the risk of EOMCI.

## 4 Discussion

This study systematically examined the combined effects and interactions of body composition and nutritional-inflammatory indices on the risk of EOMCI in patients with T2DM. The findings from K-means clustering and QGC analysis demonstrated that low body composition, accompanied by malnutrition and elevated inflammatory status, was overall associated with an increased risk of EOMCI. Subgroup analysis further revealed that the overall negative effect of body composition and nutritional inflammatory indices on EOMCI risk remained significant, particularly in males, those with longer diabetes duration, DMC, or unstable glycemic control (indicated by HbA1c in the lowest or highest tertile). Using QGC analysis and LASSO cross-validation, critical indicators with negative effects on EOMCI risk were identified, including the CALLY index, GNRI, SMMI, and BMI. In contrast, ABSI emerged as the primary indicator with a positive association with EOMCI risk. Interaction analysis further indicated that in the context of low muscle mass, malnutrition and elevated inflammatory status, as reflected by a low CALLY index, significantly exacerbated the risk of EOMCI. Similarly, the interaction between abdominal obesity, as indicated by high ABSI, and malnutrition, as indicated by low GNRI, was significant, leading to a further increase in the risk of EOMCI.

### 4.1 Sarcopenia and cognitive impairment

Recent studies have demonstrated an association between sarcopenia and cognitive impairment through cross-sectional research conducted in different populations (Chang et al., 2016; Peng et al., 2020; Komatsu et al., 2021; Hu et al., 2022; Gurholt et al., 2023). For example, Gurholt et al. (2023) utilizing data from the United Kingdom Biobank, and Hu et al. (2022) based on the China Health and Retirement Longitudinal Study (CHARLS), both confirmed this link. Additionally, systematic reviews and meta-analyses conducted by Peng et al. (2020), Chang et al. (2016) provided further evidence supporting this relationship by integrating data from multiple cross-sectional studies, with an adjusted OR of approximately 2.25. Some prospective cohort studies suggest a possible bidirectional causal relationship between sarcopenia and cognitive function. Beeri et al. (2021) demonstrated that greater baseline severity of sarcopenia increased the future risk of cognitive impairment. Conversely, a 4 years Japanese follow-up study indicated that cognitive decline could, in turn, elevate sarcopenia risk through reductions in physical and social activities (Nishimoto et al., 2024). Recent bidirectional Mendelian randomization studies by Liu et al. (2024), Lu et al. (2024) further clarified this relationship, demonstrating significant bidirectional causality between sarcopenia features, such as muscle mass, grip strength, walking speed, and cognitive function. In line with these studies, our findings revealed that low muscle mass was independently associated with an increased risk of EOMCI among young and middle-aged Chinese patients with T2DM, and was identified as a critical factor in the combined multifactorial effects analysis.

### 4.2 Association between nutritional-inflammatory status and cognitive impairment: potential advantages of the CALLY index

Various assessment tools have been utilized in different studies to investigate the relationship between nutritional status and cognitive function. For example, Yu et al. (2021) using the Mini Nutritional Assessment Short Form (MNA-SF) in a Chinese elderly population, found that after adjusting for confounders, malnutrition, anorexia, and weight loss were significantly associated with increased risks of cognitive impairment. Similarly, a Swedish cohort study indicated that individuals aged 60 and above with severe cognitive impairment had significantly elevated risks of malnutrition (Fagerstrom et al., 2011). Additionally, Lu et al. (2021) using the Elderly Nutritional Indicators for Geriatric Malnutrition Assessment (ENIGMA) based on the Singapore Longitudinal Aging Study, demonstrated that older adults with higher nutritional risk scores were more likely to develop MCI or dementia. Several studies have consistently reported that low serum albumin, an important biochemical indicator of nutritional status, is closely associated with cognitive decline. This finding has been supported by studies conducted in the Singapore Longitudinal Aging Study (Lu et al., 2021), a Chinese elderly cohort study (Ng et al., 2009), and the United States National Health and Nutrition Examination Survey (NHANES) (Hu Y. et al., 2024). The GNRI, calculated using serum albumin levels, has been widely used in nutritional assessments among older adults. A prospective study from the Chinese Longitudinal Healthy Longevity Survey (CLHLS) demonstrated a significant association between low GNRI and cognitive decline (Sun et al., 2021). However, most previous studies focused primarily on elderly populations. In the present study, we also observed a significant association between low GNRI and an increased risk of EOMCI among young and middle-aged Chinese patients with T2DM.

Elevated oxidative stress and systemic inflammation are strongly linked to cognitive impairment (Gahtan and Overmier, 1999; Takeda et al., 2014). A study based on the Atherosclerosis Risk in Communities (ARIC) cohort found that individuals in the highest quartile of systemic inflammation during middle age experienced cognitive decline approximately 7.8% faster than those in the lowest quartile (Walker et al., 2019). Chronic inflammation inhibits lymphocyte proliferation, leading to reduced lymphocyte counts and impaired secretion of anti-inflammatory cytokines; thus, low lymphocyte counts can serve as an indicator of chronic inflammation (Moro-García et al., 2018). Tsukita et al. (2021) found that low lymphocyte counts were associated with rapid declines in MoCA scores and could interact with the apolipoprotein E epsilon 4 (APOE ε4) allele to exacerbate cognitive impairment. Similarly, in patients with T2DM, low lymphocyte counts were significantly associated with cognitive decline (Du et al., 2021; Yu et al., 2023). Additionally, lower serum albumin levels were significantly associated with elevated inflammatory markers (Yamamoto et al., 2021) and mediated cognitive deterioration linked to pro-inflammatory diets (Chen et al., 2024). The PNI, calculated from serum albumin and lymphocyte count, indicated that lower PNI values, reflective of poor nutritional intake, were significantly associated with an increased risk of cognitive decline (Zhou et al., 2021). Our findings also showed a significant association between low PNI and increased risk of EOMCI in T2DM patients.

Numerous cross-sectional studies (Schram et al., 2007; Roberts et al., 2009), meta-analyses (Kuo et al., 2005; Shen et al., 2019; Leonardo and Fregni, 2023), and prospective cohort studies (Marioni et al., 2009; Arce Renteria et al., 2020; Lewis and Knight, 2021) have demonstrated that elevated CRP levels are associated with higher risks of cognitive impairment and dementia, particularly in younger populations (Lewis and Knight, 2021) and in non-memory cognitive domains (Marioni et al., 2009; Roberts et al., 2009; Arce Renteria et al., 2020). Using K-means clustering, this study similarly identified significantly lower scores in non-memory cognitive domains among participants characterized by low body composition and poor nutritional-inflammatory status. The novel CALLY index, which integrates serum albumin, lymphocyte count, and CRP levels, was assessed for the first time in this study in relation to EOMCI risk in T2DM patients. The results revealed that the CALLY index showed the strongest negative association with EOMCI risk among combined effects analyses, outperforming GNRI, PNI, and the FAR, indicating its potential utility for cognitive risk assessment.

### 4.3 Potential advantages of ABSI in identifying cognitive impairment risk

The association between BMI and cognitive impairment or dementia risk demonstrates significant heterogeneity across age, sex, and ethnicity (Cronk et al., 2010; Coin et al., 2012; Sobow et al., 2014; Vints et al., 2023). Recent meta-analyses indicated a U-shaped association between BMI and dementia risk among middle-aged populations, with both underweight and obesity increasing risk. Conversely, in elderly populations, the association is predominantly negative, where low BMI is associated with increased risk and high BMI with decreased risk (Beydoun et al., 2008; Qu et al., 2020). Our findings similarly revealed a significant association between low BMI and an increased risk of EOMCI among middle-aged patients with T2DM. However, BMI has inherent limitations as an indicator of general obesity. The American Medical Association explicitly states that BMI cannot reflect body shape differences related to ethnicity, sex, and age, nor can it adequately assess abnormal fat distribution, particularly visceral adiposity (American Medical Association, 2023). Abdominal obesity is recognized as a more critical risk factor for cognitive impairment compared to general obesity (O’Brien et al., 2020; Tang et al., 2021), although its assessment tools require further refinement. WC, despite partially reflecting abdominal obesity, cannot differentiate between subcutaneous and visceral fat and is influenced by height and body shape. The newly proposed ABSI, which integrates waist circumference, height, and weight, addresses individual variations in body shape and more accurately identifies visceral adiposity (Krakauer and Krakauer, 2012; Ji et al., 2018; Lu et al., 2023). Previous studies have demonstrated that high ABSI is independently associated with an increased risk of cognitive decline or dementia in elderly populations from the United States (Zhang Y. et al., 2024) and rural China (Wang et al., 2023). Using QGC and LASSO cross-validation analysis, our study further demonstrated that ABSI contributes significantly more to the risk of EOMCI compared to other body shape indices. This finding suggests the potential advantages of ABSI as a marker of visceral adiposity. Future prospective studies involving ethnically diverse populations are warranted to validate the clinical utility of ABSI in the early identification and stratified management of cognitive impairment.

### 4.4 Interaction of sarcopenia, nutritional-inflammatory status, and abdominal obesity in relation to cognitive impairment

Previous studies have mainly focused on the independent associations of abdominal obesity, sarcopenia, and nutritional-inflammatory status with cognitive impairment, whereas their interactions have long been overlooked. Sarcopenia often coexists with malnutrition and elevated inflammatory status (Gingrich et al., 2019; Ligthart-Melis et al., 2020; Xu et al., 2023). This coexistence might arise from protein-energy deficiencies and impaired antioxidant defenses due to malnutrition, triggering dysregulated secretion of pro-inflammatory cytokines and chronic low-grade inflammation. Chronic inflammation, in turn, activates muscle catabolic pathways and inhibits muscle synthesis (Mujico et al., 2012; Bourke et al., 2016; Xing et al., 2023), creating a vicious cycle of “malnutrition–inflammation–sarcopenia.” Cross-sectional studies conducted by Liu et al. (2021), Hu et al. (2021) in elderly community populations in western China indicated that nutritional status assessed by the MNA-SF partially mediates the relationship between sarcopenia and cognitive decline. Our study, through interaction analysis, further revealed a significant synergistic effect between low SMMI and low CALLY index on increased EOMCI risk. Moreover, the interaction between abdominal obesity (high ABSI) and malnutrition (low GNRI) further exacerbated EOMCI risk, suggesting a shared or mutually reinforcing pathological mechanisms involving visceral adiposity accumulation and nutritional-inflammatory imbalance. Visceral adiposity induces chronic low-grade inflammation through abnormal secretion of pro-inflammatory cytokines (Misiak et al., 2012; Kjærgaard et al., 2020), whereas malnutrition-induced protein-energy deficiency further weakens antioxidant defenses (Mujico et al., 2012; Bourke et al., 2016). These factors synergistically amplify inflammatory cascades. Spanish studies have highlighted the common coexistence of obesity and malnutrition in patients with acute coronary syndrome (Freeman and Aggarwal, 2020) and elderly T2DM (Sanz Paris et al., 2013). Furthermore, a prospective study by Yaffe et al. (2004) confirmed that metabolic syndrome, characterized primarily by abdominal obesity, accelerated cognitive decline only in individuals with high inflammation levels. These findings indicate inflammation as a central mediator linking metabolic abnormalities to cognitive impairment.

### 4.5 Multifactorial overlapping state in T2DM patients and its impact on cognitive function

Sarcopenia often coexists with malnutrition and chronic low-grade inflammation, especially notable when chronic diseases or cancers progress to cachexia (Evans et al., 2008; von Haehling et al., 2010; Gingrich et al., 2019; Lee et al., 2021; Ida et al., 2024; Jensen and Cederholm, 2024). Although the loss of body tissues is the core pathological feature of cachexia, high fat mass may partially mask the progressive loss of muscle and other tissue cells (Biolo et al., 2014; Gingrich et al., 2019; Livshits and Kalinkovich, 2019). Notably, sarcopenia and obesity can coexist in the early stages of chronic diseases, forming a condition termed “sarcopenic obesity.” Obesity itself may drive the onset and progression of sarcopenia through multiple mechanisms (Barazzoni and Gortan Cappellari, 2020; Schneider and Correia, 2020; Park and Choi, 2023; Booranasuksakul et al., 2024). A systematic review integrating differentially expressed microRNAs (miRNAs) in individuals with sarcopenia and obesity found that 24 miRNAs changed consistently in the same direction across 10 studies. These miRNAs are primarily involved in biological processes such as proteostasis, mitochondrial dynamics, determination of muscle fiber types, insulin resistance, and adipogenesis (Dowling et al., 2022). From a pathological perspective, systemic homeostasis imbalance caused by excessive fat accumulation is a crucial factor. Obesity-induced insulin resistance enhances skeletal muscle protein degradation via activation of the ubiquitin-proteasome system. Impaired mitochondrial oxidative phosphorylation leads to insufficient ATP production, directly reducing muscle contraction capability. Ectopic fat accumulation further exacerbates mitochondrial dysfunction and oxidative stress through lipotoxicity. Additionally, inflammatory cytokines, such as interleukin-6 (IL-6) and tumor necrosis factor-alpha (TNF-α), secreted by adipose tissue create a positive feedback loop with systemic low-grade inflammation, synergistically promoting muscle degradation. Reduced physical activity and lipid infiltration into skeletal muscle also contribute significantly to decreased muscle density and strength loss (Biolo et al., 2014; Livshits and Kalinkovich, 2019; Barazzoni and Gortan Cappellari, 2020; Park and Choi, 2023). Therefore, insulin resistance, disrupted energy metabolism, chronic inflammation, reduced physical activity, and insufficient protein intake collectively form the core mechanisms underlying the overlapping and progressive conditions of sarcopenic obesity and nutritional-inflammatory states (Biolo et al., 2014; Livshits and Kalinkovich, 2019; Barazzoni and Gortan Cappellari, 2020).

The vicious cycle involving visceral fat accumulation, chronic low-grade inflammation, insulin resistance, and pancreatic β-cell dysfunction is central to the onset and progression of T2DM. As the disease evolves, insulin resistance and β-cell dysfunction severely disrupt the body’s energy metabolism, leading to glucose and lipid homeostasis imbalance. Under normal fasting or starvation conditions, hepatic gluconeogenesis transiently activates to maintain blood glucose levels. However, in T2DM patients, this pathway remains persistently hyperactive, promoting lipolysis. When fat stores become insufficient, the body is forced to rely on protein breakdown for energy production (Aatsinki et al., 2019). This scenario results in metabolic abnormalities similar to cachexia, such as weight loss and skeletal muscle wasting (Yoshida and Delafontaine, 2015; Shih-Wei, 2022). Therefore, obesity is commonly observed in the early stages of T2DM. As the disease progresses, sarcopenia combined with obesity, accompanied by malnutrition and elevated inflammation, may develop. When energy metabolism becomes further disrupted, significant weight loss and muscle wasting become more pronounced, especially in patients with longer disease duration, multiple complications, and older age (Barazzoni and Gortan Cappellari, 2020). In the T2DM population, the overlapping conditions of sarcopenia, abdominal obesity, and malnutrition-inflammatory states are widespread (Biolo et al., 2014; Low et al., 2020; Ida et al., 2024), particularly prevalent in patients with comorbid conditions such as chronic kidney disease or heart failure (von Haehling et al., 2010; Lee et al., 2021; Ida et al., 2024). Notably, this multifactorial overlap is not limited to elderly T2DM patients; similar mechanisms may also occur in middle-aged individuals.

Cognitive impairment, T2DM, and sarcopenic obesity share common pathophysiological mechanisms, including insulin resistance, mitochondrial dysfunction, oxidative stress, and systemic inflammation (Arosio et al., 2023; Xing et al., 2023; Booranasuksakul et al., 2024). Brain insulin resistance can accelerate Alzheimer’s disease -related pathology through multiple mechanisms. Persistent peripheral hyperinsulinemia inhibits insulin transport into the brain and reduces insulin-degrading enzyme activity, resulting in impaired clearance and accumulation of amyloid-beta (Aβ) peptides. Aβ oligomers binding to neuronal insulin receptors induce synaptotoxicity, further exacerbating insulin resistance. Reduced insulin signaling also activates glycogen synthase kinase 3β (GSK-3β), promoting tau hyperphosphorylation and neurofibrillary tangle formation, creating a vicious cycle (Cholerton et al., 2013; Ma et al., 2015). Additionally, peripheral metabolic disturbances, such as hyperglycemia and hyperlipidemia, exacerbate brain energy metabolism dysfunction and suppress hippocampal function (Cholerton et al., 2013). Mitochondrial DNA mutations and metabolic disorders cause mitochondrial dysfunction and excessive reactive oxygen species (ROS) production, resulting in insufficient energy supply and organelle damage. These factors contribute to neuronal apoptosis and cognitive impairment (Mao, 2013; Lejri et al., 2019). Concurrently, chronic inflammation and impaired neurogenesis further drive neurodegeneration (Gahtan and Overmier, 1999; Xing et al., 2023). On the other hand, skeletal muscle can influence brain structure and function via the “muscle-brain axis.” In sarcopenia, dysregulated secretion of myokines with pro-inflammatory and anti-inflammatory functions (e.g., IL-6, BDNF, and irisin) impairs their beneficial effects on synaptic plasticity and neuroprotection. This dysregulation further aggravates neuroinflammation and energy deficits, ultimately accelerating cognitive decline and increasing dementia risk (Arosio et al., 2023; Xing et al., 2023). Interventions targeting improvements in insulin sensitivity and addressing mitochondrial dysfunction and oxidative stress hold promise for delaying cognitive decline and mitigating associated pathological progression.

### 4.6 Research innovations and multidimensional integrated management of high-risk populations

The innovation of this study lies in being the first to integrate muscle mass, body shape, and nutritional-inflammatory status as a comprehensive whole for evaluating their association with EOMCI risk among young and middle-aged patients with T2DM, employing multiple advanced statistical models. The results indicated that the simultaneous presence of low body weight, reduced muscle mass, abdominal obesity, malnutrition, and elevated inflammatory status significantly increased EOMCI risk. Stratified analysis demonstrated that this combined effect was statistically significant only among male patients, consistent with previous findings linking lower albumin levels (Hu Y. et al., 2024), higher CRP levels (West et al., 2020), and higher ABSI scores (Zhang Y. et al., 2024) to more pronounced cognitive decline in males. Moreover, patients with a longer disease duration, poor glycemic control, and DMC exhibited more pronounced energy metabolism disruption and oxidative stress. These patients were more likely to experience overlapping conditions, such as low muscle mass, underweight, and nutritional-inflammatory states, thus becoming high-risk groups for EOMCI. These findings provide new theoretical insights for developing effective intervention strategies, suggesting the necessity of early nutritional assessment and screening in middle-aged T2DM patients. Incorporating malnutrition and sarcopenia management into routine diabetes care, along with cognitive monitoring and interventions, could effectively reduce EOMCI risk through optimized weight management, muscle mass preservation, and comprehensive improvements in nutritional and inflammatory status.

### 4.7 Limitations and future perspectives

The study has some limitations. The cross-sectional design limits the ability to establish causal relationships, restricting findings to associations. Data from single measurements do not capture dynamic changes in body composition and nutritional inflammatory status, potentially affecting the understanding of long-term effects. Additionally, the sample’s geographic specificity may limit the generalizability of the results. Finally, potential interference from antihypertensive or statin medications on inflammatory markers such as CRP was not controlled, potentially affecting result interpretation. Future research should consider using longitudinal designs to more accurately establish causal relationships and include more diverse populations. Additionally, research on lifestyle and nutritional interventions is crucial for developing more effective targeted interventions for EOMCI in T2DM patients.

## 5 Conclusion

The study demonstrates that in patients with T2DM, the overlapping conditions of low body weight, reduced muscle mass, abdominal obesity, malnutrition, and elevated inflammatory status are overall significantly associated with an increased risk of EOMCI. This finding highlights the impact of the multifactorial interplay on cognitive health, suggesting that future interventions should focus on the integrated management of these risk factors to effectively reduce the risk of EOMCI in patients with T2DM.

## Data Availability

The raw data supporting the conclusions of this article will be made available by the authors, without undue reservation.
