# Supplementary material for: Interaction and overall effects of underweight, low muscle mass, malnutrition, and inflammation on early-onset mild cognitive impairment in type 2 diabetes
Source: Front Aging Neurosci. 2025 Mar 27;17:1498478. doi: 10.3389/fnagi.2025.1498478 (PMC11983464; doi:10.3389/fnagi.2025.1498478)
Supplement: Supplementary file 1 [file Table_1.docx]

Supplementary Material

Appendix to “Overall effects and interactions of underweight, low muscle mass, malnutrition, and inflammation on early-onset mild cognitive impairment in type 2 diabetes”

# Data collection methods and variable definitions

Healthcare professionals collected participants' sociodemographic characteristics, medical history, medication usage, and lifestyle information through standardized questionnaires, face-to-face interviews, and medical record reviews. All professionals involved underwent uniform training to ensure consistency and accuracy in data collection. Prior to the interviews, the research objectives and data usage were thoroughly explained to the participants, and informed consent was obtained. Data collection was conducted in strict accordance with the study protocol approved by the ethics committee and adhered to privacy protection regulations.

**1.1 Sociodemographic characteristics**

Sociodemographic information included sex, age, years of education, and marital status. Sex was categorized as male or female; age was recorded as the actual age at the time of the survey, in years; years of education were recorded as the number of years of formal education completed; and marital status was classified as married or unmarried (including single, divorced, or widowed).

**1.2 Lifestyle factors**

Lifestyle information included smoking status, drinking status, physical activity, and diabetes dietary control. Smoking status was categorized as current smoker, former smoker, or never smoker; drinking status was categorized as current drinker, former drinker, or never drinker. Physical activity was assessed based on whether the participant engaged in at least 150 minutes of moderate to vigorous physical activity per week, which was classified as regular exercise^1^. Diabetes dietary control was defined as adherence to dietary practices recommended by the Chinese Guidelines for the Prevention and Treatment of Type 2 Diabetes, which include a diet primarily based on grains, high in dietary fiber, low in salt, sugar, and fat, with regular meals, consistent carbohydrate distribution, and the ability to adjust diet based on blood glucose levels, as well as limiting the intake of sugary beverages^1^.

**1.3 Diabetes duration and hypoglycemia**

Diabetes duration was determined by asking participants the year they were first diagnosed with diabetes, and it was recorded in years. The assessment of hypoglycemia involved inquiring whether participants had experienced hypoglycemia in the past three months and the frequency of such episodes.

**1.4 Assessment of medical history, complications, and comorbidities**

The medical history system documented previous diagnoses across various organ systems. Healthcare professionals conducted comprehensive assessments of diabetes-related complications and comorbidities using routine examinations, including fundoscopy, diabetic neuropathy evaluation, peripheral vascular ultrasound, electrocardiography, echocardiography, and abdominal ultrasound, combined with the documented medical history. Diabetic microvascular complications were defined as the presence of diabetic nephropathy, neuropathy, or retinopathy. Peripheral arterial atherosclerosis included atherosclerosis of the carotid and lower limb arteries. Coronary heart disease (CHD) encompassed a history of angina, myocardial infarction, coronary artery bypass grafting, or stent implantation. Dyslipidemia was defined as LDL-C ≥ 160 mg/dL, HDL-C < 40 mg/dL, total cholesterol ≥ 240 mg/dL, or triglycerides ≥ 200 mg/dL^2^.

**1.5 Antidiabetic medication use**

Information on antidiabetic medication use included the types and dosages of medications that participants were currently taking. Recorded medication categories included insulin (rapid-acting, short-acting, intermediate-acting, long-acting, and premixed insulin), oral antidiabetic drugs (such as metformin, sulfonylureas, DPP-4 inhibitors, SGLT-2 inhibitors, thiazolidinediones, α-glucosidase inhibitors, and meglitinides), and GLP-1 receptor agonists. Given that metformin is recommended as the first-line oral antidiabetic drug for type 2 diabetes and that accumulating evidence suggests a significant association between metformin and cognitive function^1,3,4^, this study further categorized oral antidiabetic drugs into metformin and other oral antidiabetic drugs for group comparisons.

**1.6 Blood pressure measurement**

Blood pressure (BP) was measured using a calibrated arm-type electronic sphygmomanometer after participants had rested for at least 5 minutes. Three measurements were taken at 2-minute intervals, and the average was used as the final BP value, including systolic blood pressure (SBP) and diastolic blood pressure (DBP).

**1.7 Laboratory testing**

Participants were required to fast for at least 8 hours before venous blood samples were collected the next morning for laboratory testing. Blood cell counts and hemoglobin levels were measured using an automated hematology analyzer. A fully automated biochemical analyzer was used to measure serum albumin, fasting plasma glucose (FPG), alanine aminotransferase (ALT), aspartate aminotransferase (AST), gamma-glutamyl transferase (GGT), triglycerides (TG), total cholesterol (TC), high-density lipoprotein cholesterol (HDL-C), low-density lipoprotein cholesterol (LDL-C), and uric acid (UA). C-reactive protein (CRP) levels were measured using the rate nephelometry method. Glycated hemoglobin (HbA1c) levels were measured by high-performance liquid chromatography (HPLC). The urine albumin-to-creatinine ratio (UACR) was determined using the first morning urine sample, measured by immunoturbidimetry to assess albumin and creatinine levels, which were then used to calculate UACR. Estimated glomerular filtration rate (eGFR) was calculated based on serum creatinine levels using the modified MDRD (Modification of Diet in Renal Disease) equation.

**1.8 References**

1. Chinese Elderly Type 2 Diabetes P, Treatment of Clinical Guidelines Writing G, Geriatric E, et al. Clinical guidelines for prevention and treatment of type 2 diabetes mellitus in the elderly in China (2022 edition). *Zhonghua Nei Ke Za Zhi*. Jan 1 2022;61(1):12-50. doi:10.3760/cma.j.cn112138-20211027-00751

2. Jellinger PS, Smith DA, Mehta AE, et al. American Association of Clinical Endocrinologists' Guidelines for Management of Dyslipidemia and Prevention of Atherosclerosis. *Endocr Pract*. Mar-Apr 2012;18 Suppl 1:1-78. doi:10.4158/ep.18.s1.1

3. Ng TP, Feng L, Yap KB, Lee TS, Tan CH, Winblad B. Long-term metformin usage and cognitive function among older adults with diabetes. *J Alzheimers Dis*. 2014;41(1):61-8. doi:10.3233/JAD-131901

4. Moore EM, Mander AG, Ames D, et al. Increased risk of cognitive impairment in patients with diabetes is associated with metformin. *Diabetes Care*. Oct 2013;36(10):2981-7. doi:10.2337/dc13-0229

#
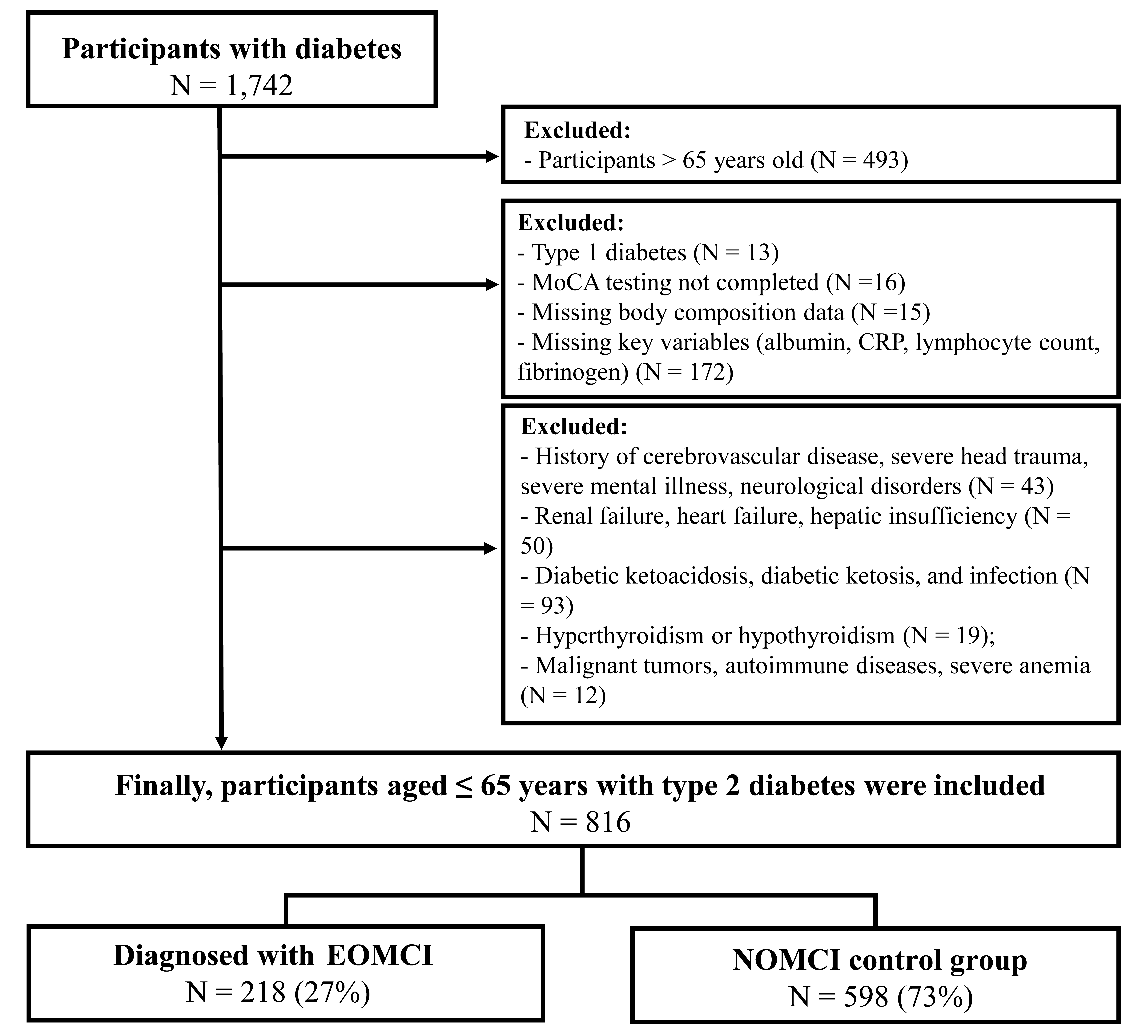
Supplementary figures

**Supplementary Figure S1. Participant selection flowchart.** Abbreviations: MoCA, Montreal Cognitive Assessment; NOMCI, non-mild cognitive impairment; EOMCI, early-onset mild cognitive impairment.

**Supplementary

Figure S2. Interaction and simple slopes analysis of body composition and nutritional inflammatory indices on the risk of EOMCI.** This figure presents the interaction effects and simple slopes analyses of body composition indices (BMI, SMMI, and ABSI) and nutritional inflammatory indices (GNRI and CALLY) on the risk of EOMCI. The left panels (A1, B1, C1, D1) depict the interaction effects across different tertiles of the body composition indices. The middle panels (A2, B2, C2, D2) show the simple slopes of the nutritional inflammatory indices on EOMCI risk at various levels of the body composition indices. The right panels (A3, B3, C3, D3) feature Johnson-Neyman plots indicating the significance regions of the nutritional inflammatory indices' effects on EOMCI risk across a range of body composition values. All analyses were conducted using generalized linear models, adjusted for covariates including age, sex, marital status, education level, diabetes duration, HbA1c, FPG, hypoglycemia frequency, smoking status, regular exercise, diabetes dietary control, UACR, AST, and Hb. Abbreviations: BMI, body mass index; SMMI, skeletal muscle mass index; ABSI, A body shape index; GNRI, geriatric nutritional risk index; CALLY, C-reactive protein-albumin-lymphocyte index; EOMCI, early-onset mild cognitive impairment; HbA1c, glycated hemoglobin; FPG, fasting plasma glucose; UACR, urine albumin-to-creatinine ratio; AST, aspartate aminotransferase; Hb, hemoglobin.
